# Autoimmune progesterone dermatitis (AIPD) triggered by intrauterine device (IUD): case report

**DOI:** 10.1186/1939-4551-8-S1-A106

**Published:** 2015-04-08

**Authors:** Ana Luiza Ribeiro Bard De Carvalho, Soloni Afra Pires Levy, Alfeu Tavares França, Rosângela Prendin Tórtora, Juliana Salvini Barbosa Martins Da Fonseca, Elisabete Da Silva Blanc, Omar Lupi

**Affiliations:** 1 Hospital Universitário Clementino Fraga Filho Hucff-Ufrj, Brazil; 2 Hospital São Zacharias, Brazil; 3 Universidade Federal Do Rio De Janeiro, Brazil

## Background

Progesterone induced dermatitis is a rare autoimmune response to endogenous progesterone that usually occurs in fertile females, in the third decade. Skin lesions occur periodically during the luteal phase of the menstrual cycle due to increase of progesterone, the symptom usually occurs 3-10 days prior to the onset of menstrual flow and resolve 2 days into menses. It may present a variety of cutaneous and mucosal manifestations, from a mild urticarial to an anaphylaxis.

## Methods

We report a case of autoimmune progesterone dermatitis (AIPD) in a woman who had placed an intrauterine device (IUD) three months before the beginning of the symptoms.

A 37-year-old woman was first seen presenting an edematous erythematous scaly pruritic eruption a few days after levofloxacin treatment for sinusitis. Skin biopsy had showed perivascular inflammatory infiltrates and frequent eosinophils and rare extravasated erythrocytes suggesting skin drug reaction. Although she had used antihistamines and high dosing corticosteroid for six months, had got any better. Patient reinforced skin lesions worsening on premenstrual period. History of IUD placement 3 months before.

## Results

After IUD removal without improvement, it was performed a progesterone intradermal skin test (IDST) with positive result. Now the patient is being treated with tamoxifen successfully.

## Conclusions

In AIPD, mechanisms by which endogenous progesterone becomes antigenic is unknown. It is proposed that previous exposure to exogenous progesterone, especially oral contraceptives or IUD, sensitize presenting cells and T helper 2 lymphocytes generate specific IgE antibodies, which cause type 1 hypersentivity. Diagnosis criteria are: (1) skin lesions related to menstrual cycle, (2) IDST positive and, (3) symptomatic improvement after inhibiting progesterone secretion by suppression ovulation. AIPD first line treatment is combined oral contraceptive, but it is also described success with GnRH agonists, tamoxifen, danazol or bilateral oophorectomy. The case report had demonstrated a woman with typical symptoms, diagnosis criteria and positive response to treatment.

## Consent

Written informed consent was obtained from the patient for publication of this abstract and any accompanying images. A copy of the written consent is available for review by the Editor of this journal.

